# AP3B1 Has Type I Interferon-Independent Antiviral Function against SARS-CoV-2

**DOI:** 10.3390/v16091377

**Published:** 2024-08-29

**Authors:** Gayatri Subramanian, Adam Hage, Friederike Feldmann, Abhilash I. Chiramel, Kristin L. McNally, Gail L. Sturdevant, Paul A. Beare, Sonja M. Best

**Affiliations:** 1 Laboratory of Virology, Rocky Mountain Laboratories, National Institute of Allergy and Infectious Diseases, National Institutes of Health, Hamilton, MT 59840, USA; 2 Laboratory of Neurological Infections and Immunity, Rocky Mountain Laboratories, National Institute of Allergy and Infectious Diseases, National Institutes of Health, Hamilton, MT 59840, USA; 3 Rocky Mountain Veterinary Branch, Rocky Mountain Laboratories, National Institute of Allergy and Infectious Diseases, National Institutes of Health, Hamilton, MT 59840, USA; 4 Research Technologies Branch, Rocky Mountain Laboratories, National Institute of Allergy and Infectious Diseases, National Institutes of Health, Hamilton, MT 59840, USA

**Keywords:** AP3B1, antiviral proteins, SARS-CoV-2, COVID-19

## Abstract

The unprecedented research effort associated with the emergence of severe acute respiratory syndrome coronavirus-2 (SARS-CoV-2) included several extensive proteomic studies that identified host proteins that interact with individual viral gene products. However, in most cases, the consequences of those virus–host interactions for virus replication were not experimentally pursued, which is a necessary step in determining whether the interactions represent pro- or anti-viral events. One putative interaction commonly identified in multiple studies was between the host adaptor protein complex 3 (AP-3) subunit B1 (AP3B1) and the SARS-CoV-2 envelope protein (E). AP3B1 is one subunit of AP-3 required for the biogenesis of lysosomal-related organelles (LROs), and its function impacts important disease processes including inflammation and vascular health. Thus, interactions between AP3B1 and SARS-CoV-2 might influence the clinical outcomes of infection. To determine if AP3B1 has a role in the SARS-CoV-2 replication cycle, we first confirmed the interaction in virus-infected cells using immunoprecipitation (IP) and immunofluorescence assays (IFA). AP3B1 is required by multiple viruses to aid in the replication cycle and therefore may be a therapeutic target. However, we found that the overexpression of AP3B1 suppressed SARS-CoV-2 replication, whereas the siRNA-mediated depletion of AP3B1 increased the release of infectious virus, suggesting an antiviral role for AP3B1. Together, our findings suggest that AP3B1 is an intrinsic barrier to SARS-CoV-2 replication through interactions with the viral E protein. Our work justifies further investigations of LRO trafficking in SARS-CoV-2 target cells and their role in viral pathogenesis.

## 1. Introduction

During the coronavirus 2019 (COVID-19) pandemic, multiple proteomic screens were conducted to better understand the protein–protein interactions between severe acute respiratory syndrome coronavirus-2 (SARS-CoV-2) and host cells. These studies had important implications for understanding the manipulation of host cellular environments during virus replication. One such study by Gordon et al. [1] used an affinity purification–mass spectrometry approach to identify host proteins interacting with individual viral proteins to identify druggable targets. Consistent with the extensive membrane remodeling within the host cell, about 40% of the host proteins interacting with viral proteins were associated with endomembrane trafficking. One such protein was the adaptor protein complex 3 subunit B1 (AP3B1) that was identified to interact with SARS-CoV-2 envelope (E) protein. This interaction was also reported in two other proteomic screens by Gordon et al. 2020 and Hoffmann et al. 2021 [2,3]. Although this interaction was commonly identified across multiple screens, it was not further characterized to understand its role in the virus replication cycle and whether it has pro- or anti-viral functions.

The adaptor complex proteins (AP-1 through AP-5) are critically involved in the intracellular trafficking of cargo proteins through endomembrane systems. Infection with SARS-CoV-2 results in a prominent modification of the endomembrane system, and in the case of beta-coronaviruses, lysosomal trafficking is utilized for viral egress and release, as opposed to a secretory pathway employed by other enveloped viruses [4]. Given the dependence of SARS-CoV-2 replication on endomembrane trafficking, we investigated the impact of AP3B1 on the production of an infectious virus. AP-3 is made up of four subunits—δ, β3, µ3 and σ3—where the β3 subunit (also known as AP3B1) is the rate-limiting factor for the formation of this complex. AP-3 mediates cargo transport from the trans-Golgi network or endosomes to lysosomes or lysosome-related organelles (LROs) [5,6]. LROs include melanosomes, platelet alpha and dense granules, lytic granules, lamellar bodies, endothelial Weibel–Palade bodies, neuronal pre-synaptic vesicles and other specialized compartments with distinct functions [7]. One such example is in melanocytes, where the AP-3 complex is crucial in the export of melanin through the Golgi to the cell membrane. Mutations in AP3B1 result in rare Hermansky–Pudlak Syndrome 2 (HPS2), and with faulty trafficking in melanocytes, one of the manifestations of this mutation is albinism. Apart from melanocytes, AP3B1 defects include prolonged bleeding due to the disrupted transport of the von Willebrand factor (VWF) in Weibel–Palade bodies (WPBs) [8], immunodeficiency, and pulmonary fibrosis due to defects in lamellar body biogenesis in type II pneumocytes. Another inflammatory defect connected to AP3B1 is hemophagocytic lymphohistiocytosis (HLH), which is marked by a cytokine storm associated with fever, rash and cytopenia [9]. Symptomatically, some overlap between HPS is also observed in patients suffering from severe COVID-19. Autopsies from COVID-19 patients revealed elevated inflammation across multiple organ systems, pulmonary fibrosis, and dysfunction in coagulation, and it was sometimes reported as a hemophagocytic lymphohistiocytosis-like disorder [10]. Whether clinical manifestations of severe COVID-19 are related to dysfunction of LROs in key cell types is unknown.

Several adaptor protein complexes (APCs) have been reported to support virus replication [11]. The AP3B1 complex is specifically reported as a required factor for the HIV-1 replication cycle, where it interacts with viral Gag protein and facilitates viral assembly and release [12]. In the case of paramyxovirus replication, AP3B1 interacts with the matrix (M) proteins of Nipah and Hendra viruses and directs the cellular trafficking necessary for viral particle formation [13]. AP-1 and AP-3 are also essential in Flavivirus replication [14]. Given the consequential roles of AP-3 in the replication of diverse pathogens, we focused on validating the interaction between SARS-CoV-2 E protein and AP3B1 and aimed to understand the role of AP3B1 during the infection cycle of SARS-CoV-2.

## 2. Materials and Methods

### 2.1. Cells and Virus

A549 cells expressing ACE2 were used for experiments measuring virus replication [15]. The virus strain used for this study was SARS-CoV-2 WA1 (GenBank: MN985325.1) propagated in Vero E6 cells (ATCC, Manassas, VA, USA, CRL-1586). Cells were infected with indicated MOI and incubated for 30 min, followed by a PBS wash and the replacement of the tissue culture media (in complete Dulbecco’s Modified Eagle Medium [DMEM] containing 10% fetal bovine serum [FBS; Thermo Fisher, Waltham, MA, USA], penicillin and streptomycin). HEK293 (ATCC, Manassas, VA, USA, CRL-1573) was used for the co-immunoprecipitation assays. Infected sample supernatants were collected and analyzed for infectious virus using plaque assays, and values are presented as plaque forming units/mL (PFU/mL). Plaque assays were performed by carrying out ten-fold serial dilutions of collected supernatants, in triplicate and inoculated on Vero cells, in 48-well tissue culture plates. After 30 min of incubation, cells were overlayed with 1.5% carboxymethyl cellulose, followed by incubation at 37 °C for 3 days. After this, cells were fixed in 10% formalin for 1 h, and plaques were counted after staining with 1% crystal violet prepared in 10% ethanol. All infections with SARS-CoV-2 were performed under biosafety level 3 (BSL3) conditions.

### 2.2. Declaration for SARS-CoV

All infections with SARS-CoV (Tor) were performed under biosafety level 4 (BSL-4) conditions at the Rocky Mountain Laboratories Integrated Research Facility (Hamilton, MT, USA). Standard operating protocols for working with SARS-CoV and removing samples from BSL-4 conditions were approved by the Rocky Mountain Laboratories Institutional Biosafety Committee.

### 2.3. siRNA Treatment

Small interfering RNA (siRNA) against AP3B1 (sc-41165) and AP3D1 (sc-60176) were procured from Santa Cruz Biotechnology (SCBT, Dallas, TX, USA) and transfected using RNAiMax using the manufacturer’s transfection protocol (Thermo Fisher, Waltham, MA, USA). Control siRNA A (SCBT sc-60176) was used a control.

### 2.4. Plasmids and Transfection

SARS-CoV-2 E protein cDNA was a kind gift from Nevan Krogan (University of California, San Francisco, CA, USA). It was cloned into a pDEST 3X Flag (C-terminal) tagged plasmid using a Gateway LR Cloning System. AP3B1 cDNA (Genecopoeia, Rockville, MD, USA) was cloned into a pENTR plasmid followed by introduction into pDEST HA (N terminal) using the Gateway LR Cloning system (Thermo Fisher, Waltham, MA, USA). A flag tag on the C-terminus of the SARS-CoV-2 E protein and an HA tag on the C-terminus of human AP3B1 were introduced using the LR cloning system. pUC19 was used as a transfection control. Plasmids were transfected into cells using the Lipofectamine LTX with PLUS reagent transfection reagent (Thermo Fisher, Waltham, MA, USA) following the manufacturer’s protocol. Cell viability was measured using the MTT Cell Proliferation Assay kit (ATCC, Manassas, VA, USA) according to the manufacturer’s instructions.

### 2.5. Immunofluorescence Assay and Quantification

A549 (ACE2) cells were seeded on glass coverslips, and after the indicated times post viral adsorption or transfection, they were fixed in 10% formalin. Cells were permeabilized using 0.1% Triton-X and 0.1% Na-citrate solution for 5 min followed by blocking with 10% normal goat serum for 1 h. The primary antibodies were SARS-CoV-2 E (Novus Biologicals, Centennial, CO, USA, #NBP3-25646), J2 (English & Scientific Consulting, Budapest, Hungary, #10010200), AP3B1 (SCBT, Dallas, TX, USA, #sc-517083), HA (Cell Signaling Technology, Danvers, MA, USA, #3724), and Flag (Cell Signaling Technology, Danvers, MA, USA, #14793) (all used at 1:250 dilution, except dsRNA/J2 used at 1:1000 dilution); secondary antibodies (Alexa Fluor Plus 488, Alexa Fluor Plus 555, used at 1:1000 dilution, Thermo Fisher, Waltham, MA, USA) were prepared in 1% normal goat serum; and cellular nuclei were stained using DAPI. Slowfade Diamond was used as mounting medium. Images were acquired using a Zeiss 710 and analyzed using ZEN version 2.6 (Blue edition) software (Zeiss, Oberkochen, Germany).

### 2.6. Interferon Treatment

A549 cells were treated with 100 units/mL of IFNβ (PBL Assay Science, Piscataway, NJ, USA, 11410) and incubated for 24 h. Cells were then harvested for analysis by Western blot or qRT-PCR.

### 2.7. Western Blot Analysis

Cells were lysed with a radioimmunoprecipitation buffer (Sigma Aldrich, St. Louis, MO, USA, R0278, 50 mM Tris-HCl, pH 8.0, with 150 mM sodium chloride, 1.0% Igepal CA-630 (NP-40), 0.5% sodium deoxycholate, and 0.1% sodium dodecyl sulfate) containing a phosphatase inhibitor (PhosSTOP, Roche, Vienna, Austria) and a protease inhibitor (cOmplete Protease Inhibitor Cocktail, Roche, Vienna, Austria) and prepared to a final concentration of 1× LaemmIisample buffer containing beta-mercaptoethanol. HEK293 cells were transfected with plasmids (at 1 μg for every 1 × 10^6^ cells) and harvested 24 h after transfection in (RIPA). Lysates were prepared in a final concentration of 1× LaemmIi sample buffer containing beta-mercaptoethanol. RIPA buffer had additional inhibitors—complete protease inhibitor and phosphatase inhibitor (Roche, Vienna, Austria). The prepared samples were run on gradient polyacrylamide gels (BOLT Bis-Tris gels 4–12%, Thermo Fisher, Waltham, MA, USA) and transferred using semi-dry transfer (BioRad, Hercules, CA, USA) to PVDF membranes before being probed using the indicated primary and secondary antibodies. The primary antibodies used were AP3B1 (Proteintech, Rosemont, IL, USA # 13384-1-AP), SARS-CoV Spike S1/S2 (Genetex, Irvine, CA, USA # GTX632604), Actin (Sigma-Aldrich, St. Louis, MO, USA, Cat #A2228), HA (Cell Signaling Technology, Danvers, MA, USA, #3724, #2367), Flag (Sigma-Aldrich, St. Louis, MO, USA, #F1804, Cell Signaling Technology, Danvers, MA, USA, #14793), and ISG15 (Thermo Fisher, Waltham, MA, USA, MA5-29371; 1:2000). The secondary antibodies used were goat anti-mouse (Thermo Fisher, Waltham, MA, USA, #31430) and goat anti-rabbit (Thermo Fisher, Waltham, MA, USA #31430). Blots were incubated with a primary antibody for 16 h at 4 °C, and a secondary antibody was incubated for 1 h at room temperature. Pierce ECL Western Blotting substrate and Super Signal West Femto substrate (Thermo Fisher, Waltham, MA, USA) were used to detect protein expression. An iBright Imaging system was used for visualization (Thermo Fisher, Waltham, MA, USA).

### 2.8. qRT-PCR Primers

Total RNA was extracted and subject to reverse transcription using the SuperScript VILO cDNA synthesis kit (Thermo Fisher). Real-time qPCR was performed in 384 plates in triplicate using iTaq Universal SYBR Green Supermix and analysed using a QuantStudio Pro (Thermo Fisher, Waltham, MA, USA). The following primers were used to amplify mRNA: AP3B1 (Origene, Rockville, MD, USA: Cat# HP207142) Fwd: GTGCCTCAATGGCTTGGTCTGT; Rev: GTGATACTGTCCAGGAGTTTGGC. ISG15 (Origene: Cat# HP208303) Fwd: CTCTGAGCATCCTGGTGAGGAA; Rev: AAGGTCAGCCAGAACAGGTCGT. IFITM1 Fwd: TGACCATTGGATTCATCCTG; Rev: TGCACAGTGGAGTGCAAAG. h18S Fwd: GTAACCCGTTGAACCCCATT; Rev: CCATCCAATCGGTAGTAGCG. Gene expression was normalized to 18S by the comparative CT method (ΔΔCT).

### 2.9. Statistical Analysis

Data from virus titrations was analyzed by Mann–Whitney U non-parametric tests or ANOVA, with *p* < 0.05 considered statistically significant. GraphPad Prism 10, Version 10.2.0, was used for these analyses. Colocalization analysis was performed using Zen software version 2.6 (Zeiss, Oberkochen, Germany).

## 3. Results

### 3.1. The Host Protein AP3B1 Interacts with SARS-CoV-2 Envelope (E) Protein

**Figure 1:**
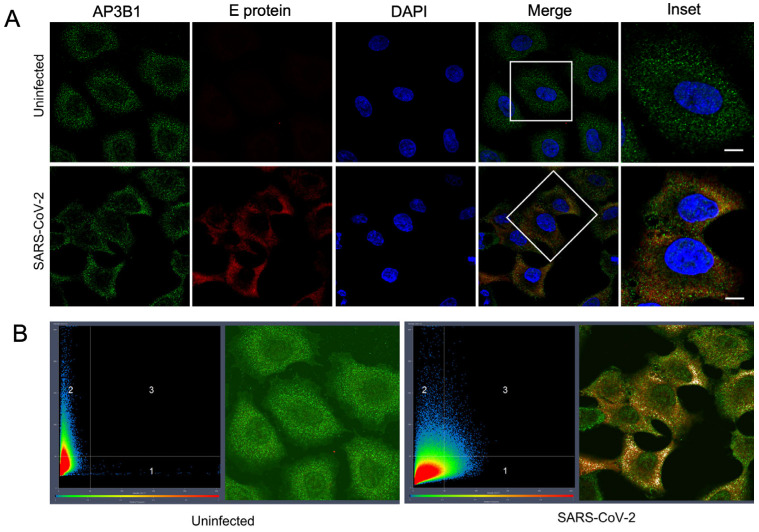
AP3B1 interacts with SARS-CoV-2 E. (**A**) A549 (ACE2) cells were infected with SARS-CoV-2 (MOI 1) and stained for endogenous AP3B1 (green) and SARS-CoV-2-E (red), revealing colocalization (yellow) of the two proteins. Scale bar represents 10 μM. Representative image shown from two independent experiments. White box depicts area shown in Inset. (**B**) Colocalization analysis of endogenous AP3B1 in uninfected A549 cells (green; Quadrant 2 of left panel) or cells infected with SARS-CoV-2 counterstained for the viral Envelope (E) protein (red; Quadrant 1 of right panel). Pixels where colocalization is apparent in Quadrant 3 are pseudo-colored white.

**Figure 2:**
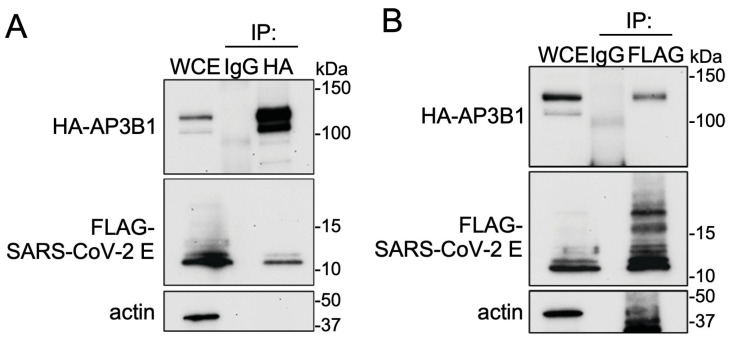
AP3B1 interacts with SARS-CoV-2 E following ectopic expression. (**A**) HEK293 cells were transfected with Flag-SARS-CoV-2-E and HA-AP3B1. Co-immunoprecipitation of Flag-SARS-CoV-2-E was observed when HA-AP3B1 was subjected to affinity purification. Representative images shown from three independent experiments. (**B**) Co-immunoprecipitation of HA-AP3B1 following affinity purification of Flag-SARS-CoV-2 E. Representative images shown from three independent experiments. WCE, whole cell extract.

A putative interaction between human AP3B1 and the SARS-CoV-2 E protein has been identified in three previous proteomic studies using ectopically expressed viral gene products [1,2,3]. To confirm the interaction during infection, we infected A549 cells (human epithelial cells) stably expressing human ACE2 with a WA-1 SARS-CoV-2 strain. An immunofluorescence assay (IFA) staining for endogenous AP3B1 and the viral E protein revealed colocalization in the small cytosolic puncta (Figure 1A). Quantifying the number of colocalizing pixels (Quadrant 3) divided by the pixels in Quadrants 1 and 3 that were positive for E using Zen software suggested that 23.6 ± 9.2% of E was also positive for AP3B1 (mean ± SD over four fields) (Figure 1B). To test if the colocalization represents a physical interaction, co-immunoprecipitation was performed following the expression of plasmids encoding HA-AP3B1 and SARS-CoV2-E-FLAG in HEK293 cells. Immunoprecipitation using either anti-HA or anti-FLAG antibodies resulted in the co-immunoprecipitation of a reciprocal protein (Figure 2A,B). Taken together, these results confirm the published interactions between AP3B1 and the SARS-CoV-2 envelope protein.

### 3.2. Overexpression of AP3B1 Inhibits SARS-CoV-2 Replication

**Figure 3:**
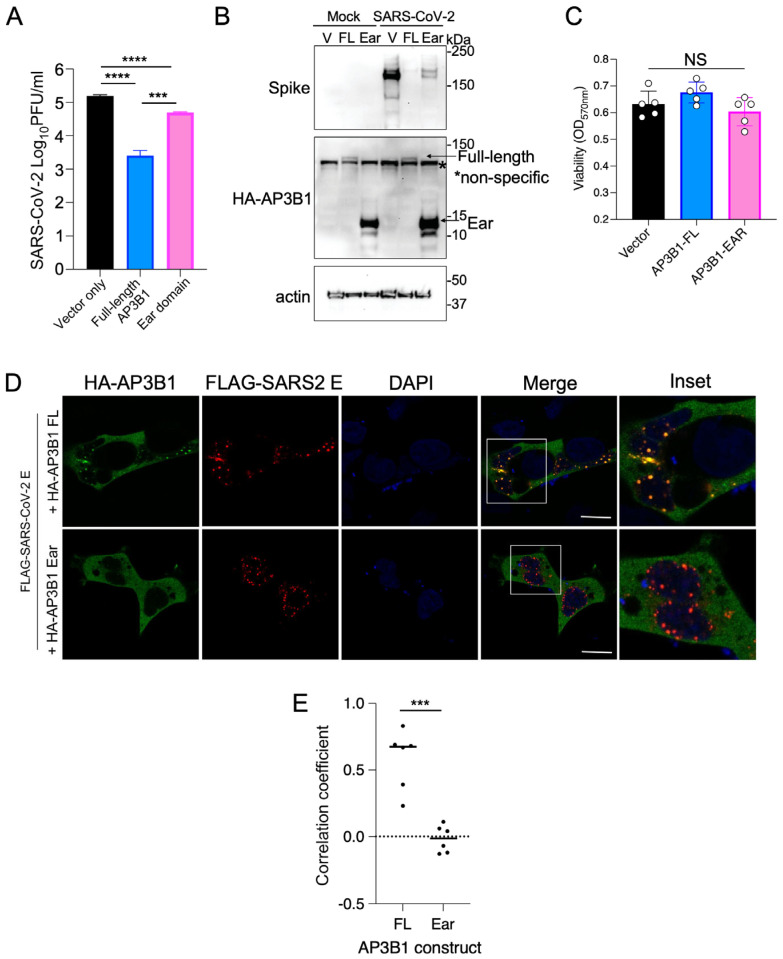
AP3B1 ear domain is insufficient for inhibition of SARS-CoV-2. (**A**) A549 cells expressing human ACE2 were transfected with plasmids encoding full-length AP3B1 or the ear domain of AP3B1 only and infected with SARS-CoV-2 for 48 h. Release of infectious virus was measured by plaque assay. (**B**) Samples from (**A**) were analyzed by Western blotting for SARS-CoV-2 spike protein expression and (**C**) viability by MTT assay. Representative images shown from two independent experiments. (**D**) Immunofluorescence analysis of colocalization between full-length HA-AP3B1 (top panel) or HA-AP3B1 ear domain (bottom panel) and SARS-CoV-2 E (red). Scale bar represents 10 μM. Representative images shown from two independent experiments. White box depicts area shown in Inset. (**E**) Quantification of correlation coefficient from two independent experiments; data points represent individual cells. Error bars represent mean ± SD with statistics from one-way ANOVA with (**A**) Tukey’s post test or (**B**) Sidak’s post test. Data in (**E**) analyzed by two-way *t*-test. NS, not significant, *** *p* < 0.0005, **** *p* < 0.0001.

To test whether AP3B1 supports or inhibits viral replication, we ectopically expressed the HA-tagged full-length (FL) AP3B1 in ACE2-expressing A549 cells followed by infection with SARS-CoV-2. Supernatants were harvested 48 h post infection (hpi). The expression of full-length AP3B1 reduced the release of infectious virus particles by approximately 100-fold (Figure 3A), associated with a greatly reduced expression of the spike protein, as a marker of virus replication (Figure 3B). We then sought to begin understanding which domain of AP3B1 was associated with the suppression of SARS-CoV-2 replication. AP3B1 has three domains—the head (1–642 aa), hinge (643–809 aa), and ear (810–1092 aa)—with the ear domain responsible for cargo binding in the cell. Unfortunately, the cDNAs of the isolated head and hinge domains did not express detectable protein. However, the expression of the ear domain was successful and could be used to determine its effects on virus replication. In this case, expression of the AP3B1 ear domain still exhibited some inhibitory activity but was significantly impaired in its ability to reduce SARS-CoV-2 replication compared to the full-length protein, as measured by both infectious virus titers and spike protein expression at 48 hpi. Cell viability was not changed following the expression of full-length AP3B1 or the ear domain (Figure 3C). By IFA, the co-expression of the FL AP3B1 protein together with the SARS-CoV-2 E protein resulted in co-localization in distinct puncta formed by E, while the expression of the AP3B1 ear domain did not strongly co-localize (Figure 3D,E). These results suggest that AP3B1 is inhibitory to SARS-CoV-2 replication, potentially through interactions with the viral E protein, but the AP3B1 cargo binding domain does not mediate those interactions in isolation from the head and/or hinge domain.

### 3.3. Endogenous AP3B1 Inhibits Replication of SARS-CoV-2 but Not SARS-CoV

**Figure 4:**
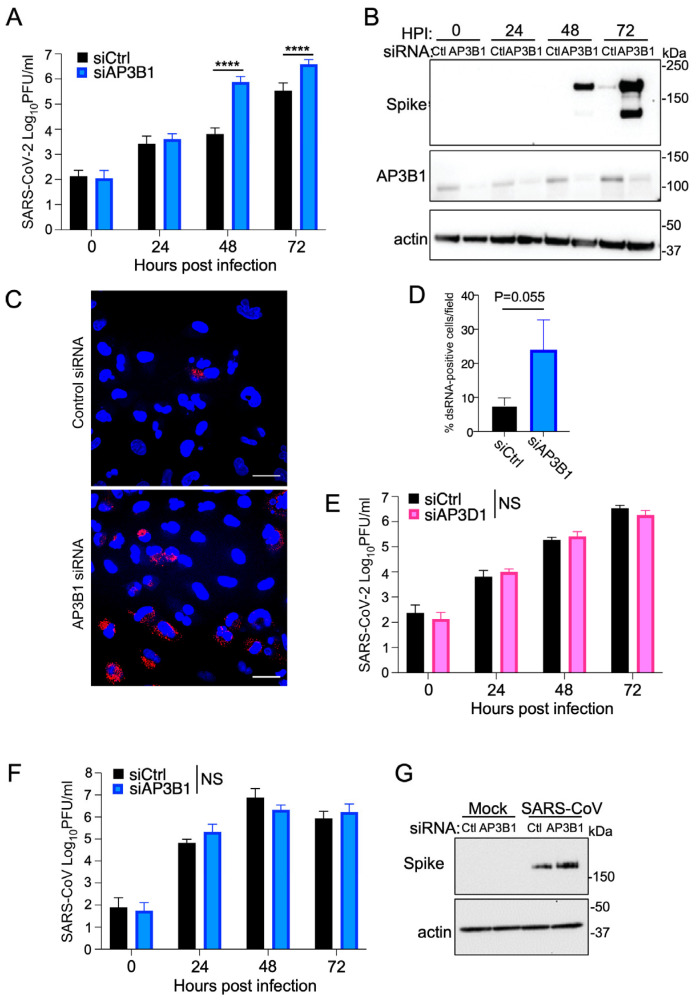
AP3B1 is antiviral for SARS-CoV-2, not SARS-CoV. (**A**) A549 cells expressing human ACE2 were treated with siRNA against AP3B1 or a scrambled control, and infected with SARS-CoV-2. Virus replication was monitored over time by plaque assays of infectious virus in cell supernatants. **** *p* < 0.0001 by one-way ANOVA with Tukey’s post test. (**B**) Samples from (**A**) were analyzed by Western blotting for SARS-CoV-2 spike protein expression and AP3B1 protein levels. Representative image shown from three independent experiments. (**C**) Immunofluorescence staining for viral dsRNA (red) in SARS-CoV-2-infected A549 cells expressing human ACE2 treated with siRNA against AP3B1 or a scrambled control. Scale bar represents 30 μM. DAPI staining of nuclei is shown in blue. (**D**) Quantification of (**C**) across four fields and analyzed by standard *t*-test. (**E**) A549 cells expressing human ACE2 were treated with siRNA against AP3D1 or a scrambled control, and infected with SARS-CoV-2. Virus replication was monitored over time by plaque assays of infectious virus in cell supernatants from three experiments. NS, not significant measured by one-way ANOVA. (**F**) A549 cells expressing human ACE2 were treated with siRNA against AP3B1 or a scrambled control, and infected with SARS-CoV. Virus replication was monitored over time by plaque assays of infectious virus in cell supernatants from three experiments. NS, not significant measured by one-way ANOVA. (**G**) Samples from (**F**) were analyzed by Western blotting for SARS-CoV spike protein expression. All error bars represent mean ± SD.

To test the antiviral effect of AP3B1 in a reciprocal approach, we used siRNA to reduce endogenous protein levels and tested the effect of this treatment on virus replication. Cells were infected with an MOI of 0.1, and virus replication was measured by plaque assay over time. The depletion of AP3B1 resulted in between a 10- and 100-fold increase in the release of an infectious virus at 48 and 72 hpi compared to cells that received control siRNA treatment (Figure 4A), as well as an increase in the expression of viral spike protein at these timepoints (Figure 4B). Using IFA to stain for dsRNA as a replicate intermediate produced during viral replication, we observed increased numbers of cells positive for dsRNA when AP3B1 was depleted (Figure 4C,D). To test the specificity of the antiviral function of AP3B1, we compared the effect of reducing expression of another component of the AP3C—AP3D1. Here (Figure 4E), the siRNA-mediated depletion of AP3D1 made no difference to viral titers. To understand whether the antiviral effect of AP3B1 was broadly applicable to closely related coronaviruses, we used the siRNA-based knockdown approach to infect A549 cells with SARS-CoV and measured infectious virus released over time. The infectious titers of SARS-CoV showed no differences between the cells that received control siRNA and siRNA for AP3B1 (Figure 4F), and spike protein expression was not reduced (Figure 4G), suggesting that the antiviral activity of AP3B1 is specific for SARS-CoV-2.

### 3.4. AP3B1 Is Not an Interferon-Stimulated Gene (ISG)

**Figure 5:**
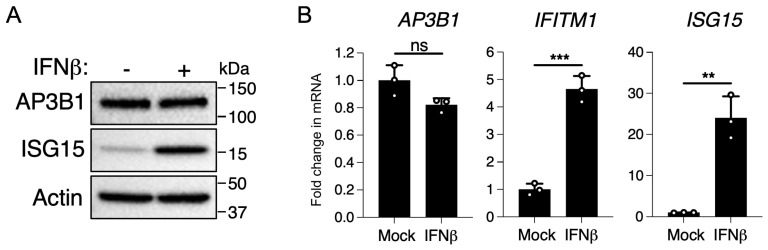
AP3B1 is not interferon inducible. (**A**) A549 cells expressing human ACE2 were treated with 100 units/mL of IFNβ and analyzed for ISG15 and AP3B1 protein expression or (**B**) expression of AP3B1, IFITM1, and ISG15 mRNA normalized to 18s ribosomal RNA. Increased expression of ISG15 and IFITM1 confirms IFNβ stimulation of treated cells. Error bars represent mean ± SD; statistics performed by unpaired *t*-test; ns = not significant, ** *p* < 0.005, *** *p* < 0.0005.

Our findings thus far indicate that AP3B1 is a novel antiviral protein that has specificity for SARS-CoV-2. As the expression of many antiviral proteins is responsive to type I interferons, we tested whether AP3B1 is an interferon-inducible antiviral protein. However, the stimulation of A549 cells with IFNβ did not result in an upregulation of AP3B1 expression by protein or mRNA, in contrast to ISG15 when used as a positive control for protein and *ISG15* and *IFITM1* mRNA when used as positive controls for gene expression (Figure 5A,B). Thus, AP3B1 is not an interferon-inducible antiviral protein in A549 cells, suggesting that it represents a steady-state intrinsic barrier to SARS-CoV-2 replication.

## 4. Discussion

Adaptor protein complexes facilitate the sorting of transmembrane and cargo proteins to specific membrane compartments within the cell. The AP-3 complex facilitates cargo sorting from the trans-Golgi network to lysosomes and LROs. In this study, we identified AP3B1 as a host-cell restriction factor for SARS-CoV-2 through interactions with the viral E protein. As a structural protein, E is a component of the coronavirus virion, along with M (membrane), S (spike) and N (nucleocapsid). The assembly of SARS-CoV-2 is coordinated at Golgi membranes, where the structural proteins M and E are necessary for viral budding. Viral egress from the cell is through deacidified lysosomes [4], the deacidification of which is mediated through E and ORF3a [16,17]. Consistent with this finding, E is only a minor component of coronavirus virions, suggesting that E functions in virion maturation also involve non-particle-associated interactions with the host cell. Recently, Pearson et al. showed that the Golgi-to-lysosome trafficking of E is dependent on adaptor protein-1 (AP-1) [18]. Once at the lysosome, E functions as a viroporin to enable pH neutralization and the repurposing of the lysosome for virion transport. Failure to deliver E for lysosome pH neutralization results in a reduced replication of SARS-CoV-2 [18]. AP-3 can also traffic cargo from the trans-Golgi network to lysosomes or LROs, and adaptor protein complexes recognize similar conserved motifs in cargo proteins [5]. Thus, the inhibition of SARS-CoV-2 replication by AP3B1 suggests that AP-3 may compete against the pro-viral role of AP-1 in the trafficking of E to divert it to non-productive sites. Future work will examine this hypothesis.

The finding that AP3B1 has antiviral functions against SARS-CoV-2 contrasts with the known roles of AP-3 in HIV-1 and paramyxovirus replication, where AP3B1 is essential for virus assembly and release in both cases [12,13,19]. Within a HIV-1-infected cell, the Gag polyprotein directs the virus assembly process. Viral budding occurs predominantly at the plasma membrane in T cells but can occur at multivesicular bodies (MVBs) in macrophages. Gag interacts directly with the δ subunit of the AP-3 complex to aid in its trafficking to the late endosome/MVB, where it may acquire ESCRT components for budding [20]. This trafficking process appears to require interactions between Kif3a, a motor protein of the kinesin superfamily, and AP3B1 [12]. In support of a role for AP3B1 in this process, HIV-1 particle assembly is defective in fibroblasts derived from HPS2 patients [19]. Interestingly, in a genome-wide association study, AP3B1 single nucleotide polymorphisms were found to have significant association with HIV-1 subtype C acquisition in populations in Botswana or were highly associated with AIDS progression in a United States–European American cohort [21]. Thus, interactions between the AP3 complex and viral proteins associated with particle trafficking and assembly have the potential to be highly consequential to viral pathogenesis or transmission in humans.

The further elucidation of the consequences of SARS-CoV-2 E interactions with AP3B1 for AP3 function are needed. The importance of germline variants of AP3B1 as monogenetic causes of human inflammatory (HLH) or immunodeficiency (HPS2) syndromes suggests that the mislocalization of AP3-dependent cargo in SARS-CoV-2-infected cells could have consequences for the pathogenesis of COVID-19. In the lung, the loss of AP-3-mediated trafficking of lamellar bodies in surfactant-producing alveolar type 2 cells contributes to a gain of function that results in the enhanced activation of repair pathways associated with pulmonary fibrosis in HPS2 [22], which is also a key feature of severe COVID-19. Furthermore, elevated levels of von Willebrand factor have been observed in the serum of patients with severe COVID-19. von Willebrand factor is made by endothelial cells or megakaryocytes and stored in WPBs that are exocytosed in an AP-3-dependent manner [8]. Both endothelial cells and megakaryocytes have been shown to be infected by SARS-CoV-2 [23]. Thus, the dysregulation of WPBs may contribute to endothelial cell dysfunction, hypercoaguability and thrombosis [24]. Importantly, germline variants in AP3B1 have been associated with the development of severe cytokine profiles and fatal outcomes in COVID-19 [25], suggesting that AP-3 and LRO trafficking in the context of infection warrants further investigation.

## Data Availability

Data is available on request.
